# The Modulation of PPAR*γ*1 and PPAR*γ*2 mRNA Expression by Ciglitazone in CD3/CD28-Activated Naïve and Memory CD4+ T Cells

**DOI:** 10.1155/2012/849195

**Published:** 2012-04-02

**Authors:** Mohd Nor Norazmi, Rafeezul Mohamed, Asma Abdullah Nurul, Nik Soriani Yaacob

**Affiliations:** ^1^School of Health Sciences, Universiti Sains Malaysia, Kelantan, 16150 Kubang Kerian, Malaysia; ^2^School of Dental Sciences, Universiti Sains Malaysia, Kelantan, 16150 Kubang Kerian, Malaysia; ^3^School of Medical Sciences, Universiti Sains Malaysia, Kelantan, 16150 Kubang Kerian, Malaysia

## Abstract

Given their roles in immune regulation, the expression of the nuclear receptor peroxisome proliferator-activated receptor *γ* (PPAR*γ*) 1 and 2 isoforms was investigated in human naïve (CD45RA+) and memory (CD45RO+) CD4+ T cells. Stimulation of both types of cells via the CD3/CD28 pathway resulted in high expression of both PPAR*γ* receptors as measured by real-time PCR. Treatment with the PPAR*γ* agonist, ciglitazone, increased PPAR*γ*1 expression but decreased PPAR*γ*2 expression in stimulated naïve and memory cells. Furthermore, when present, the magnitude of both PPAR*γ* receptors expression was lower in naïve cells, perhaps suggesting a lower regulatory control of these cells. Similar profiles of selected proinflammatory cytokines were expressed by the two cell types following stimulation. The induction of PPAR*γ*1 and suppression of PPAR*γ*2 expressions in naïve and memory CD4+ T cells in the presence of ciglitazone suggest that the PPAR*γ* subtypes may have different roles in the regulation of T-cell function.

## 1. Introduction

Peripheral CD4+ T cells can be divided into two broad functional groups based on their expression of distinct isoforms of the CD45 surface molecule, CD45RA representing naïve CD4+ T cells and CD45RO representing memory CD4+ T cells [1]. Memory CD4+ T cells require a shorter lag time to proliferate when they are stimulated by antigens and are less dependent on costimulation than are naïve CD4+ T cells [2]. On the other hand, naïve CD4+ T cells have been reported to be the source of autoreactive lymphocytes in multiple sclerosis [3, 4], suggesting a differential regulatory mechanism for these cells.

The peroxisome proliferator-activated receptors (PPARs) are ligand-activated receptors that belong to the nuclear receptor superfamily [5]. Three isoforms of PPARs have been identified and are encoded by separate genes, namely, PPAR*α*, *γ*, and **β*/*δ** [6, 7]. PPAR*γ* is predominantly expressed in adipose tissue, colon, spleen, adrenal gland, and monocytes/macrophage [6, 7]. This isoform is further divided into four subtypes: PPAR*γ*1, *γ*2, *γ*3, and *γ*4 due to alternative promoter use and RNA splicing [8]. PPAR*γ*1, PPAR*γ*3, and PPAR*γ*4 encode for the same protein product, while the PPAR*γ*2 protein contains an additional 28 amino acids at its N-terminus. PPAR*γ* ligands include the naturally occurring arachidonic acid metabolite, 15-deoxy-D12,14-prostaglandin J2 (15d-PGJ2), as well as the thiazolidinedione (TZD) group of drugs such as ciglitazone and certain novel non-TZD insulin-sensitizing agents [9, 10].

PPAR*γ* expressed in murine T-cells plays a regulatory role in T-cell activation [11]. Previous experiments showed that murine helper-T-cell clones and freshly isolated splenocytes express PPAR*γ*1 but not PPAR*γ*2 mRNA and that 15d-PGJ2 and ciglitazone inhibited the proliferative responses and IL-2 production of these cells when stimulated with the specific antigen and anti-CD3 antibodies, respectively [11]. Similarly, it was reported that 15d-PGJ2 and troglitazone suppressed IL-2 production of PHA-stimulated peripheral blood T cells [12]. PPAR*γ* has been shown to physically bind to the transcription factors AP-1 and NFAT [12, 13], which regulate the IL-2 promoter thus blocking their binding to the promoter and hence inhibiting the transcription of the IL-2 gene.

These studies indicate an important immunoregulatory role for PPAR*γ* in T-cell function. It will, therefore, be interesting to investigate whether naïve and memory CD4+ T cells behave in the same manner with regard to the expression of PPAR*γ* and whether their activation modulate the expression of the PPAR*γ* receptor differently. It would also be important to explore the impact on cytokine expression in these T-cell subsets upon activation of PPAR*γ*, in particular selected proinflammatory cytokines, which are important in autoreactivity such as autoimmune diabetes [14].

Most studies on the role of PPAR*γ* have used semi-quantitative measurements to assess the mRNA level of the receptor. Since subtle changes in PPAR*γ* levels may result in significant changes to various downstream events as postulated by other types of receptor-signaling molecules [15], an accurate quantification of PPAR*γ* isoform levels following cellular activation would need to be carried out.

We propose to study the expression of PPAR*γ*1 and PPAR*γ*2 in unstimulated and stimulated naïve and memory CD4+ T-cell subsets using quantitative real-time PCR. To further dissect the role of PPAR*γ*1 and PPAR*γ*2 in immune activation, the PPAR*γ* agonist, ciglitazone, was used to modulate the activation status of these cell types and assess the modulation of their expression levels as well as those of selected proinflammatory cytokines in these cells.

## 2. Materials and Methods

### 2.1. Isolation of Naïve and Memory CD4+ T Cells from Peripheral Blood

Peripheral blood collection has prior approval from the Universiti Sains Malaysia Ethics Committee and collected after informed consent was obtained. Human naïve and memory CD4+ T cells were isolated from the peripheral blood by immunomagnetic separation. Briefly, blood was obtained from normal donors, and the peripheral blood mononuclear cells (PBMCs) were isolated by the Ficoll gradient centrifugation and incubated with a panel of biotin-conjugated monoclonal antibodies against CD8, CD14, CD16, CD19, CD36, CD56, CD123, TCR*γδ*, and glycophorin A (Miltenyi Biotec, Germany). CD45RA and CD45RO microbeads were added reciprocally for the negative isolation of memory and naïve CD4+ T cells. The purity of the isolated naïve and memory CD4+ T cells were generally 90–95% as determined by flow cytometric analysis.

### 2.2. In Vitro Stimulation of Naïve and Memory CD4+ T Cells

Naïve and memory CD4+ T cells were suspended at 2 × 10^5^ cells/mL in complete RPMI 1640 medium (10% FBS, 100 U/mL penicillin, and 100 *μ*g/mL streptomycin) containing CD3/CD28 beads at a 1 : 1 cell/bead ratio in 25 cm^2^ tissue culture flasks. Twenty *μ*M of ciglitazone solution was added when required at day 0 of culture. This concentration of ciglitazone was determined based on the minimum concentration required to cause a reduction in cell proliferation as reported in the literature [11, 13, 16]. The flasks were incubated for 5 days in a humidified incubator at 37°C in 5% CO_2_.

### 2.3. Proliferation Assay

Naïve and memory CD4+ T cells were suspended in 200 *μ*L of complete RPMI 1640 medium at a concentration of 1 × 10^3^/well in triplicate wells of a 96-well flat-bottom plate and stimulated with CD3/CD28 beads for 5 days as previously described [17]. When required, ciglitazone (20 *μ*M) was added at day 0 of culture. Ten *μ*L of diluted [^3^H] thymidine (1 *μ*Ci) was added to each well at 0, 24, 48, 72, and 96 h after stimulation. After incubation for another 20–22 h, the cells were harvested to represent day 1, 2, 3, 4, and 5, respectively, using the Innotech cell harvester system (Innotech AG, Switzerland). The incorporation of [^3^H] thymidine into DNA was quantified using a liquid scintillation counter by Hidex data analysis software (Hidex, USA).

### 2.4. Total RNA Extraction and cDNA Synthesis

Total RNA was extracted from unstimulated and stimulated naïve and memory CD4+ T cells with or without ciglitazone treatment using the RNeasy Mini kit (Qiagen, USA) and QIAshredder (Qiagen, USA) according to the manufacturer's instructions. Briefly, the cells were lysed in RLT buffer and the beads were depleted using Dynal MPC. The lysed cells were applied onto the QIAshredder column followed by the RNeasy Mini spin column after addition of 70% ethanol. The sample column was then centrifuged, and the flow-through discarded before 700 *μ*L of RW1 buffer was added into the column. Following centrifugation, the mixture was washed twice in 500 *μ*L RPE buffer before 50 *μ*L of RNase free water was added into the column to dissolve the total RNA. The RNA was eluted by centrifugation, and its integrity was assessed by gel electrophoresis while RNA purity and concentration were measured by spectrophotometry (Biophotometer, Eppendorf, Germany).

Total RNA (between 0.5 to 5 *μ*g) was reverse transcribed into cDNA using the RevertAid H Minus first strand cDNA synthesis kit (MBI Fermentas, USA) in the presence of 0.5 *μ*g oligo(dT)_18_ primer in nuclease-free deionized water. The mixture was firstly incubated at 70°C for 5 minutes. The reaction mixture was then mixed with 4 *μ*L of 5x reaction buffer, 20 unit ribonuclease inhibitor, and 2 *μ*L of 10 mM dNTP mix, followed by incubation at 37°C for 5 minutes. The process of reverse transcription was performed at 42°C for 1 hour using 200 unit of RevertAid H Minus M-MuLV. Finally the process was terminated by heating at 70°C for 10 minutes. The success of cDNA synthesis was confirmed by running a PCR using human *β*-actin primer (Maxim Biotech, USA).

### 2.5. Competitive Real-Time PCR

The PPAR*γ*1 gene was amplified and quantified using the following primers/probe: forward primer 5′-CTT TAT GGA GCC CAA GTT TGA GTT-3′; reverse primer 5′-GGC TTC ACA TTC AGC AAA CCT-3′ and TaqMan probe 5′-TGC CAA GTC GCT GTC ATC TAA TTC CAG TG-3′. The PPAR*γ*2 gene was amplified and quantified using the following primers/probe: forward primer 5′-GGG TGA AAC TCT GGG AGA TTC TC-3′; reverse primer 5′-GAT GCC ATT CTG GCC CAC-3′ and TaqMan probe 5′-TGA CCC AGA AAG CGA TTC CTT CAC TGA-3′. A total volume of 22.5 *μ*L master mix, which included the TaqMan Universal Master Mix (ABI, USA), TaqMan probe, forward and reverse primers, and sterile distilled water was added in each well of the PCR plate prior to the addition of 50 ng of target cDNA. The master mix contains a dye (ROX) for normalization. Five dilutions of internal standards (plasmids containing the PPAR*γ*1 and PPAR*γ*2 genes) were chosen from the range of 10^−4^ pmol to 10^−8^ pmol. For nontemplate control (NTC) wells, only water was added. The reaction plate was sealed with an optical adhesive cover, centrifuged briefly to avoid any bubbles, and placed in the real-time PCR apparatus to begin the reaction. All samples were run in triplicates.

The reaction was initiated at 50°C for 2 min. This step was required for optimal AmpErase uracil-N-glycosylase (UNG) enzyme activity to decontaminate any DNA carryover. The temperature was increased to 95°C for 10 min to activate the AmpliTaq Gold enzyme. This was followed by 45 cycles of denaturation at 95°C for 15 sec and primer annealing and extension stages at 60°C for 1 min each.

### 2.6. Multiplex PCR (MPCR)

The expression levels of TGF*β*, IL-1*β*, IL-8, TNF*α*, GM-CSF, and IL-6 were measured in unstimulated and stimulated naïve and memory CD4+ T cells with or without treatment with ciglitazone using the MPCR kit for Human Inflammatory Cytokines Genes Set-1 (Maxim Biotech, USA). The expression of the house-keeping gene, GAPDH, was used for normalization. The MPCR was carried out according to the manufacturer's instructions. Briefly, 1x MPCR buffer, 1x MPCR primer mix, 2.5 units of Taq polymerase, and 0.1 *μ*g cDNA template were mixed in a 50 *μ*L reaction; the optimum annealing temperature for the MPCR analysis was 66°C and subjected to 35 cycles of PCR, with denaturing, annealing, and extension temperatures at 94, 58, and 70°C for 1 min each, respectively. Following MPCR, the products were fractionated electrophoretically in a 2% agarose gel containing 0.5 *μ*g/mL ethidium bromide and analysed by the Image Master Total Lab v1.00 (Amersham Pharmacia, USA). 

### 2.7. Statistical Analysis

The profiles of [^3^H] thymidine incorporation of naïve and memory CD4+ T cells after *in vitro* stimulation with or without ciglitazone treatment were compared and analysed using the Kruskal-Wallis test. The PPAR*γ*1 and PPAR*γ*2 expression and cytokine profiles of unstimulated and stimulated naïve and memory CD4+ T cells with or without ciglitazone treatment were compared and analysed using the Mann-Whitney *U* test by statistical program for social science (SPSS) version 11.0 computer program (SPSS Inc., USA).

## 3. Results

### 3.1. Proliferative Response of CD3/CD28-Stimulated Naïve and Memory CD4+ T Cells

**Figure 1:**
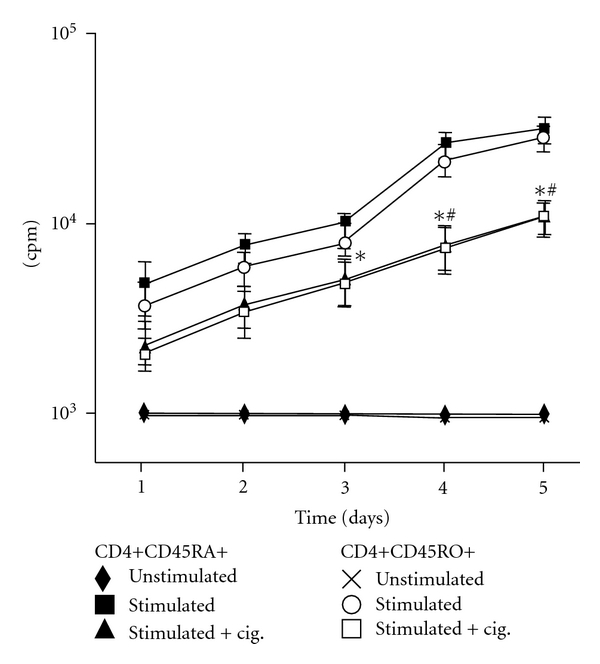
Proliferation assay. [^3^H] thymidine incorporation of naïve (CD45RA+) and memory (CD45RO+) CD4+ T cells following *in vitro* stimulation with CD3/CD28 beads, in the presence or absence of ciglitazone. Data are expressed as the mean cpm of triplicate cultures ± SEM. The experiments were repeated three times. Statistical analyses were performed using the Kruskal-Wallis test.*P < 0.05 (for naïve CD4+ T cells) or ^#^ *P* < 0.05 (for memory CD4+ T cells) of ciglitazone-treated, compared to untreated stimulated cells.

The proliferative response of purified naïve and memory CD4+ T cells following *in vitro* stimulation with CD3/CD28 was assessed. Anti-CD3/CD28 enhanced proliferation in both naïve and memory CD4+ T cells as depicted by the incorporation of [^3^H] thymidine (Figure 1). From day 1 to 5 after stimulation, the cell proliferation rate increased by more than 20-fold. There was no significant difference in the proliferation rate between the naïve and memory CD4+ T cells. The addition of ciglitazone decreased the degree of proliferation in naïve and memory CD4+ T cells by about 10-fold. Ciglitazone significantly decreased the proliferation rate of activated naïve CD4+ T cells on days 3, 4, and 5 (*P* < 0.05) and that of activated memory CD4+ T cells on days 4 and 5 (*P* < 0.05).

### 3.2. Quantification of PPAR*γ*1 and PPAR*γ*2

**Figure 2:**
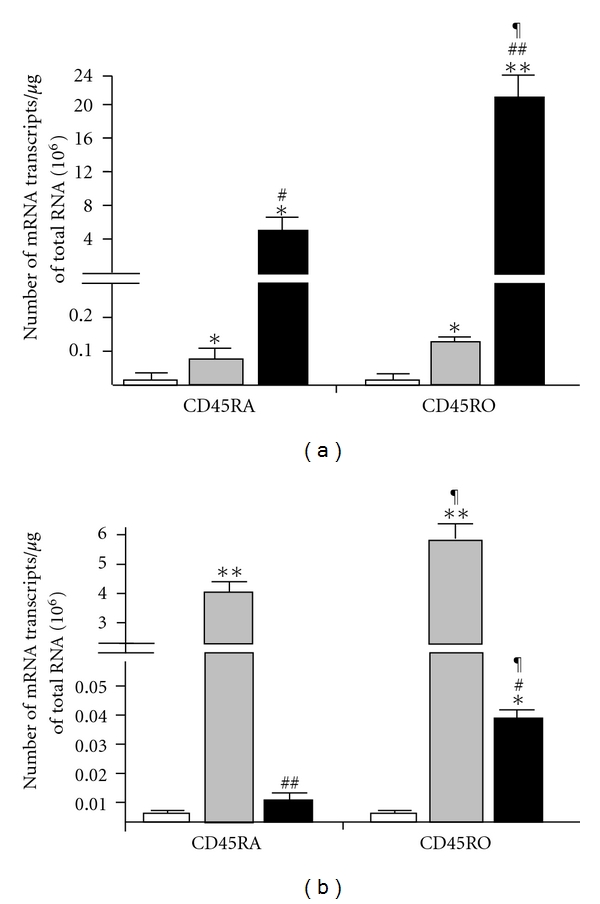
PPAR*γ*1 and PPAR*γ*2 mRNA expression. (a) PPAR*γ*1 and (b) PPAR*γ*2 gene expression levels in unstimulated (open bar, *n* = 13) and stimulated (grey bar, *n* = 13) naïve and memory CD4+ T cells or those treated with ciglitazone (solid bar, *n* = 8). The PPAR*γ*1 and PPAR*γ*2 gene expression levels were calculated as the number of mRNA transcripts per *μ*g total RNA. The data plotted is the mean mRNA transcripts ± SEM. Statistical analyses were performed using the Mann-Whitney *U* test. *P < 0.05; **P < 0.01—significantly different from unstimulated cells. ^#^ *P* < 0.05; ^##^ *P* < 0.01—significantly different from CD3/CD28–stimulated cells. ^¶^ *P* < 0.05—significantly different from correspondingly treated CD45RA+ cells.

Unstimulated naïve and memory CD4+ T cells expressed low constitutive levels of PPAR*γ*1 mRNA, whereas stimulated naïve and memory CD4+ T cells expressed significantly higher levels of the receptor in both cell types (average of 7 × 10^4^ and 1.2 × 10^5^ mRNA transcripts/*μ*g of total RNA, for naïve and memory CD4+ T cells; resp., *P* > 0.05; Figure 2(a)). Stimulated memory CD4+ T cells displayed higher PPAR*γ*1 expression than naïve CD4+ T cells (*P* < 0.05). Ciglitazone treatment significantly increased the expression of PPAR*γ*1 by about 70-fold and 160-fold in naïve and memory CD4+ T cells (*P* < 0.01), respectively. PPAR*γ*1 expression remained significantly higher in stimulated memory compared to stimulated naïve CD4+ T cells in the presence of ciglitazone (*P* < 0.01).

Unstimulated naïve and memory CD4+ T cells expressed 10-fold lower constitutive levels of PPAR*γ*2 mRNA compared to PPAR*γ*1 (Figure 2(b)). Stimulated naïve and memory CD4+ T cells express very high levels of PPAR*γ*2 mRNA in both cell types (average of 3.9 × 10^6^ and 5.5 × 10^6^ mRNA transcripts/*μ*g of total RNA, in naïve and memory CD4+ T cells, resp.). PPAR*γ*2 expression in stimulated memory CD4+ T cells expressed higher levels of the receptor compared to naïve CD4+ T cells. In contrast to PPAR*γ*1, the addition of ciglitazone significantly decreased the expression of PPAR*γ*2 by about 470-fold and 150-fold in naïve and memory CD4+ T cells, respectively (*P* < 0.01). However, after treatment with ciglitazone, PPAR*γ*2 expression was significantly higher in stimulated memory compared to stimulated naïve CD4+ T cells (*P* < 0.01).

**Figure 3:**
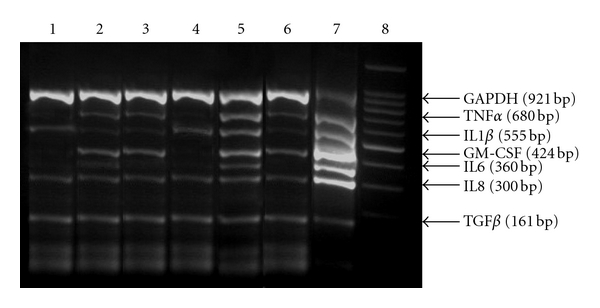
Example of multiplex PCR readout of inflammatory cytokine gene expression. Lane 1: unstimulated naïve CD4+ T cells. Lane 2: stimulated naïve CD4+ T cells. Lane 3: stimulated naïve CD4+ T cells + ciglitazone. Lane 4: unstimulated memory CD4+ T cells. Lane 5: stimulated memory CD4+ T cells. Lane 6: stimulated memory CD4+T cells + ciglitazone. Lane 7: positive control. Lane 8 : 100 bp marker.

**Figure 4:**
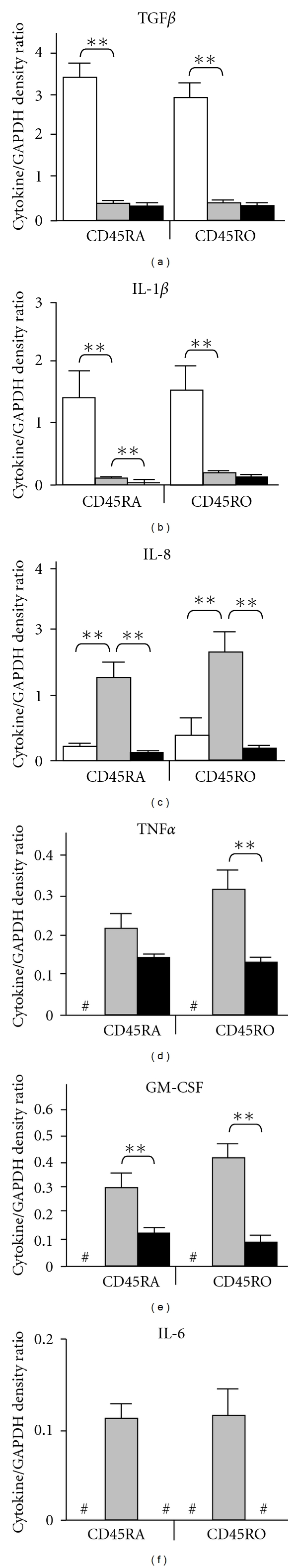
Inflammatory cytokine expression. Relative mRNA expression levels of selected cytokine genes in naïve and memory CD4+ T cells following CD3/CD28 stimulation in the presence or absence of ciglitazone (*n* = 5). Untreated cells (open bar), stimulated cells (grey bar), and ciglitazone-treated cells (solid bar) were assessed for their relative expression of (a) TGF*β*, (b) IL-1*β*, (c) IL-8, (d) TNF*α*, (e) GM-CSF, and (f) IL-6, as a ratio of GAPDH. Statistical analyses were performed using the Mann-Whitney *U* test. *P < 0.05, **P < 0.01. ^#^Significance levels cannot be analyzed because the gene expression was not detectable.

Figure 3 shows an example of a gel electrophoresis of the MPCR products of selected inflammatory cytokines in unstimulated and stimulated naïve and memory CD4+ T cells with or without ciglitazone treatment. The expression of various cytokines was compared by densitometric analyses and expressed as a ratio of GAPDH. The results were then plotted as histograms as depicted in Figure 4.

As shown in Figure 4(a), the expression levels of TGF*β* gene were higher in unstimulated naïve and memory CD4+ T cells but decreased significantly in their stimulated state (*P* < 0.01). The addition of ciglitazone did not significantly alter the expression of TGF*β* in both stimulated cells. IL-1*β* gene expression was also higher in unstimulated naïve and memory CD4+ T cells but decreased significantly in their stimulated state (*P* < 0.01). Ciglitazone further decreased the expression of IL-1*β* in stimulated naïve (*P* < 0.01) but not in stimulated memory CD4+ T cells (Figure 4(b)). IL-8 gene was expressed at low levels in unstimulated naïve and memory CD4+ T cells but significantly increased in both cell types upon activation (*P* < 0.01). IL-8 expression decreased in memory and naïve CD4+ T cells to its unstimulated states upon addition of ciglitazone (*P* < 0.01) (Figure 4(c)).

Figure 4(d) shows the *de novo* TNF*α* expression in stimulated naïve and memory CD4+ T cells. There was no significant difference in the expression of TNF*α* in both cell types after activation. Ciglitazone significantly decreased the expression of TNF*α* in stimulated memory (*P* < 0.01) but not in naïve CD4+ T cells. GM-CSF was also expressed in stimulated naïve and memory CD4+ T cells but not in their unstimulated state. There was no significant difference in the expression of GM-CSF in both cell types after activation. GM-CSF expression was significantly reduced in stimulated naïve and memory CD4+ T cells in the presence of ciglitazone (*P* < 0.01; Figure 4(e)). Figure 4(e) shows that only stimulated naïve and memory CD4+ T cells expressed IL-6. The addition of ciglitazone completely abolished the expression of IL-6 in both stimulated cells. The results clearly show *de novo *expression of TNF-*α*, GM-CSF, and IL-6 upon activation of naïve and memory CD4+ T cells.

## 4. Discussion

It is now established that PPAR*γ* is involved in the regulation of T-cell function, as well as macrophage and dendritic cell activities [18–20]. In view of the fact that human naïve and memory CD4+ T cells differ in the requirements for activation and magnitude of their cellular responses [21] and autoreactivity [3, 4], we investigated the effect of the PPAR*γ* agonist, ciglitazone, on the mRNA expression of PPAR*γ*1 and PPAR*γ*2 and on a number of inflammatory cytokines produced by these cells. No previous studies on the expression of PPAR*γ*1 and PPAR*γ*2 in human naïve and memory CD4+ T cells have been reported.

Consistent with previous reports [11, 13, 16, 20], ciglitazone treatment resulted in a tenfold reduction in the proliferative response of both CD3/CD28-stimulated naïve and memory CD4+ T-cell subsets. Inhibition of proliferation in activated naïve T cells by PPAR*γ* agonists, such as ciglitazone, has been previously attributed to apoptosis [16], although whether this occurs via a PPAR*γ*-dependent or independent pathway remains to be elucidated.

Using RT-PCR, PPAR*γ*1 and PPAR*γ*2 were found to be highly expressed in both naïve and memory CD4+ T cells upon activation through the TCR and costimulatory CD28 pathway. Consistent with previous findings [21], only low expression levels of both transcripts in unstimulated CD4+ T cells were recorded. Interestingly, previous studies reported that PPAR*γ* is constitutively expressed in human peripheral blood mononuclear cells [6, 22]. However, this may be due to its expression by other cell subsets in the mononuclear cell population such as monocytes [18], B cells [23], and NK cells [24].

It is interesting to note the low level expression of PPAR*γ*1 and PPAR*γ*2 in resting human naïve and memory CD4+ T cells. This may suggest that their roles are primarily in the regulation of responding T cells. It is also noteworthy that higher levels of both transcripts are found in activated memory CD4+ T cells as opposed to their low level expression in activated naïve T cells, suggesting that regulation of memory CD4+ T cells may require higher-level expression of PPAR*γ* compared to naïve CD4+ cells.

Treatment with ciglitazone enhanced the expression of PPAR*γ*1 but greatly diminished that of PPAR*γ*2 in both the naïve and memory CD4+ T cells. Previous studies have reported that PPAR*γ* agonists such as troglitazone [12] and pioglitazone [22] attenuated the expression of the receptor. Here, we report that ciglitazone enhances the expression of PPAR*γ*1 but greatly diminishes the expression of PPAR*γ*2 in both naïve and memory CD4+ T cells. This apparent discrepancy can be attributed to the fact that the above studies did not distinguish between the two PPAR*γ* isoforms. PPAR*γ*1 can be regarded as a “subset” of PPAR*γ*2 which contains additional 28 amino acids at its N-terminus. Thus, measuring PPAR*γ* expression without distinguishing the two isoforms may not provide an accurate reflection of the receptor's role in immune regulation. The lack of specific antibodies against PPAR*γ*1 has however impeded our attempt to differentiate the protein expression of these receptors in the current study. The decrease in PPAR*γ*2 expression cannot be attributed to cell death via apoptosis [16] since the expression of PPAR*γ*1 was enhanced and that the cell recovery after 5 days was above 90% (results not shown).

The different roles played by the two PPAR*γ* isoforms in CD4+ T-cell regulation can be inferred from their expression levels displayed at pre- and posttreatment with ciglitazone. Thus, although the fold increase in PPAR*γ*2 expression was higher than that observed for PPAR*γ*1, it was almost completely abrogated upon addition of ciglitazone. A previous report [12] showed that troglitazone and 15d-PGJ2 inhibited IL-2 production in the PPAR*γ*2-expressing but not in PPAR*γ*2-nonexpressing transfected Jurkat T cells, suggesting that PPAR*γ*2 is involved in regulating T cell function. The almost complete abrogation of PPAR*γ*2 expression following treatment with ciglitazone is interesting and requires further investigations, such as inhibition studies. The present lack of specific chemical inhibitors for PPAR*γ*2, however, would complicate such studies for the time being.

As mentioned above, activation of PPAR*γ* by its ligands has been shown to induce apoptosis in T cells [16, 25]. Hence the question arises whether cells that express higher levels of PPAR*γ*2 are more prone to apoptosis, resulting in the preferential “elimination” of PPAR*γ*2-expressing cells. Single-cell analyses, including the measurement of PPAR*γ*1 and PPAR*γ*2 protein levels, should be carried out to address these questions. However, as anti-PPAR*γ*1 antibodies are not available, such experiments may prove currently challenging. It will also be important to investigate the molecular regulation of PPAR*γ*1 and PPAR*γ*2 promoters in order to understand the possible differential control of their expression.

Since differential expression of PPAR has been shown to correlate with selected cytokine production [26, 27] and that naïve and memory CD4+ T cells may play a differential role in autoimmunity [3, 4], the level of various proinflammatory cytokines that were expressed in the resting and activated naïve and memory CD4+ T cells with or without treatment with ciglitazone was subsequently determined. While TGF*β*, IL-8, and IL-1*β* expression in resting naïve and memory CD4+ T cells has previously been reported [28], their expression in activated naïve and memory CD4+ T cells has not been previously studied.

Activated naïve and memory CD4+ T cells displayed low expression levels of both TGF-*β* and IL-1*β*, further reduced upon stimulation with ciglitazone (in the case of IL-1*β*, further reduction was only observed in activated naïve CD4+ T cells). These findings are in agreement with those previously reported [13, 19]. Unstimulated naïve and memory CD4+ T cells displayed low levels of IL-8 which significantly increased upon activation. However, the addition of ciglitazone dramatically reduced IL-8 expression. This observation is in contrast to a previous finding that 15d-PGJ2, another PPAR*γ* ligand, induced the expression of IL-8 in human T cells via a PPAR*γ*-independent manner [29]. Thus there may be distinct response against different ligands with regard to the function of these receptors. Future studies will, therefore, need to include the use of several PPAR*γ* ligands to determine the detailed mechanistic roles of the receptors in immune response.

Activation of both naïve and memory CD4+ T cells induced *de novo* expression of TNF*α*, GM-CSF, and IL-6, whereas treatment of these activated cells with ciglitazone diminished TNF*α* and GM-CSF expression, and totally abrogated IL-6 expression. Previous studies showed significant reduction in the release of LPS-stimulated TNF*α* upon activation of placental, amnion, and choriodecidual, tissues with both 15d-PGJ2 and troglitazone [30]. Ciglitazone, troglitazone, and 15d-PGJ2 also inhibited RSV-induced release of TNF*α* in A549 epithelial cells [31]. As previously reported [19], the expression of GM-CSF in activated naïve and memory CD4+ T cells may play a role in inducing the expression of PPAR*γ*1 and PPAR*γ*2 in both activated cells. Reduction of GM-CSF expression after ciglitazone treatment has also been reported in mast cells where a PPAR*γ* agonist decreased the antigen-induced GM-CSF production [32].

The present observation that IL-6 is produced in similar levels by both naïve and memory CD4+ T cells has previously been reported [33]. IL-6 plays an essential role in activating naïve and memory CD4+ T cells through the CD2 molecule [34]. Unlike naïve T cells, CD4 memory T cells can undergo proliferation when stimulated with anti-CD2 in the absence of APCs since they are able to use self-produced IL-6 [35]. However, the current study shows that activation of naïve CD4+ T cells via the CD3 and CD28 pathways also induced the production of IL-6. This may have occurred through the engagement of the CD28 molecule which may act by amplifying the activation signals in an autocrine fashion.

A previous report [36] supports our observation that ciglitazone completely abolished the expression of IL-6 in activated naïve and memory CD4+ T cells. There is also evidence that chronic IL-6 treatment suppressed the expression of PPAR*γ* [26], and the suppression of PPAR*γ* functions resulted in excessive production of the cytokine [37]. The mechanism through which ciglitazone affects cytokine production remains to be elucidated. There is evidence [11, 12, 19] to suggest that this may occur through activation of transcription factors such as AP-1, STAT-1, and NF-*κ*B. Since there are no reports to suggest that the *cis*-element of inflammatory cytokine genes contains PPAR*γ* binding site, inhibition may occur indirectly via transrepression as described above [13]. It was also reported that 15d-PGJ2 treatment rendered I*κ*B resistant to degradation upon cellular activation [38], hence, preventing NF-*κ*B activation. However, since ciglitazone is structurally different from 15d-PGJ2, the mechanism of inhibition of NF-*κ*B and AP-1 activity by ciglitazone may differ from its inhibition by 15d-PGJ2.

## 5. Conclusions

PPAR*γ*1 and PPAR*γ*2 have differential regulatory roles in responding naïve and memory CD4+ T cells. Overall, naïve CD4+ T cells seem to be more sensitive to PPAR*γ* activation, although further studies need to be carried out to confirm this observation. The availability of specific antibodies and specific antagonists against these two isoforms is needed to enable a more precise elucidation of their purported differential functions in T-cell regulation. In addition, the precise mechanism of how PPAR*γ*1 and PPAR*γ*2 regulate the response of naïve and memory cells or the immune response in general will require further investigations utilizing single-cell analytical tools.
